# Evaluation of Antiviral Activity of *N*‑Phenyl-2-Phenoxyacetamides Derived from Carvacrol against Mayaro Virus: *In Vitro* Analyses and *In Silico* Insights

**DOI:** 10.1021/acsomega.5c05194

**Published:** 2025-11-20

**Authors:** Bianca Muniz Lacerda Ventura, Olivia Géraldine Audrey Avome Nguema, Rayane Luiza de Carvalho, Patrícia Fontes Pinheiro, Alícia da Conceição Terbutino da Silva, Sara Andrade Machado de Godoy, Bárbara Braga Ferreira, Christiane Mariotini-Moura, Raphael de Souza Vasconcellos, Sérgio Oliveira de Paula, Leandro Licursi de Oliveira, Michelle Dias de Oliveira Teixeira

**Affiliations:** † Department of Biochemistry and Molecular Biology, Federal University of Viçosa, Avenida Peter Henry Rolfs, s/n, 36570-900 Viçosa, MG, Brazil; ‡ Department of Chemistry, Federal University of Viçosa, Avenida Peter Henry Rolfs, s/n, 36570-900 Viçosa, MG, Brazil; § Department of Medicine and Nursing, Federal University of Viçosa, Avenida Peter Henry Rolfs, s/n, 36570-900 Viçosa, MG, Brazil; ∥ Department of General Biology, Federal University of Viçosa, Avenida Peter Henry Rolfs, s/n, 36570-900 Viçosa, MG, Brazil

## Abstract

Mayaro virus (MAYV), a member of the *Togaviridae* family and *Alphavirus* genus, is an emerging and neglected arbovirus that causes Mayaro fever, which can be severely debilitating disease. Although it is responsible for sporadic outbreaks in forested areas of Latin American countries, MAYV has the potential to emerge in an urban transmission cycle, as mosquitoes of the *Aedes* genus are capable of transmitting the virus, potentially leading to epidemics. Despite this threat, there are currently no licensed vaccines or antiviral drugs available, highlighting the urgent need for effective antiviral compounds. In this study, the antiviral activity of eight *N*-phenyl-2-phenoxyacetamide derivatives of carvacrol against Mayaro virus was evaluated. Among the tested compounds, compound **9** demonstrated the greatest potential, preserving 97% of cell viability following viral infection. Plaque reduction assays revealed that compound **9** interacts directly with the viral particle, interfering with the adsorption and internalization steps of the viral replication cycle. This hypothesis was further supported by *in silico* analyses, which demonstrated the compound’s ability to interact with regions of the viral glycoproteins E1 and E2 involved in receptor binding and membrane fusion. Taken together, these findings highlight compound **9** as a promising candidate for the development of effective antiviral therapies against Mayaro virus.

## Graphical abstract

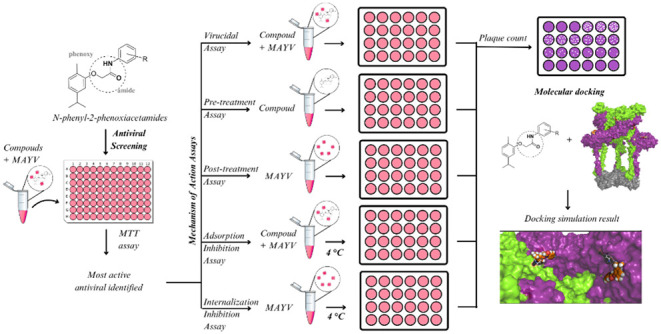

## Introduction

The Mayaro virus (MAYV) is a neglected arbovirus endemic to the forested regions of Central and South America, primarily within the Amazon Basin.^1–3^ The virus was first isolated in 1954 in Mayaro County, Trinidad, from the blood of patients presenting with a short-term febrile illness^4^ and has since caused several outbreaks throughout Latin American countries.^5^ It is currently estimated that 58.9 million people are at risk of infection, with 46.2 million residing in Brazil, which accounts for the highest number of reported cases.^6^

Infection in humans is responsible for Mayaro fever, an arthritogenic disease whose main symptoms include high fever, maculopapular rash, ocular pain, myalgia, and arthralgia. Moreover, severe complications such as myocarditis, hemorrhagic manifestations, and neurological disorders may occur.^7,8^ Although the acute disease is generally self-limiting, a significant proportion of patients experience persistent or recurrent chronic polyarthralgia for months following infection, which can be severely debilitating.^9,2^

MAYV belongs to the genus *Alphavirus*, family *Togaviridae*, and is part of the Semliki antigenic complex, a serological group also comprising other alphaviruses such as Bebaru virus (BEBV), Chikungunya virus (CHIKV), Getah virus (GETV), Semliki Forest virus (SFV), Ross River virus (RRV), O’nyong–nyong virus (ONNV), and Una virus (UNAV), all of which share antigenic sites.^10,11^ This antigenic sharing results in cross-reactivity in serological assays, complicating diagnosis.^12,13^ Consequently, it is believed that the number of reported Mayaro fever cases is underestimated and that the true burden imposed by MAYV remains largely unknown.^14,15^

MAYV is an enveloped icosahedral virus with a positive-sense single-stranded RNA genome comprising two open reading frames (ORFs) that encode the nonstructural proteins (nsP1, nsP2, nsP3, nsP4) forming the replicase complex, and the structural proteins (C, E1, E2, E3, 6K).^16,17^ Viral entry into cells occurs via the endosomal pathway, mediated by the interaction between the viral glycoprotein E2 and the adhesion molecule MXRA8, identified as the cellular receptor for MAYV and other alphaviruses.^5,18–20^ Conformational changes in the E1 glycoprotein induced by the low pH of the endosome trigger the fusion of the endosomal membrane with the viral envelope, leading to the release of the viral genome into the cytoplasm, where replication occurs.^16^

In recent years, concern regarding the emergence of MAYV infections has increased.^2,21^ The virus is maintained in nature through an enzootic cycle involving *Haemagogus* mosquitoes and animal reservoirs, with most human infections occurring in forested or nearby areas.^6,22–24^ However, there is growing concern with respect to the urbanization of MAYV, which, combined with the lack of prior immunity in the population, could lead to epidemics.^25–27^ The competence of *Aedes* mosquitoes for virus transmission,^28,29^ combined with homology to CHIKV, the occurrence of Mayaro fever cases near tropical cities where *A. aegypti* is endemic, and the detection of the virus in travelers and migratory birds, could contribute to the establishment of an urban transmission cycle.^11^ Additionally, a case of aerosol transmission in a laboratory setting has been reported.^30^ Thus, MAYV represents a threat to public health.^3,7,31^

Nevertheless, there are currently no vaccines or antiviral treatments available for the prevention or treatment of Mayaro fever. Although several vaccines have been developed in recent years, none are licensed for human use.^32–34^ Management is limited to symptomatic relief, with analgesics, nonsteroidal anti-inflammatory drugs, and antipyretics, which do not exhibit antiviral activity. Given the potential for MAYV to mimic the epidemiological progression of CHIKV, which could overwhelm healthcare systems in many countries, the search for specific compounds is urgent.^7,25,27,31^

Natural products are a valuable source of antiviral compounds.^31^ Carvacrol, a phenolic monoterpene present in oregano (*Origanum vulgare* L.), thyme (*Thymus vulgaris*), pepper (*Lepidium flavum*), wild bergamot (*Citrus aurantium* var. *bergamia* Loisel), and other plants, exhibits antiviral activity against various viruses, including HIV-1, hepatitis A virus, different noroviruses, herpes simplex virus type-2, and pseudorabies virus,^35–40^ while *in silico* analyses suggest that it may also have potential as an antiviral agent against SARS-CoV-2.^41^ In addition, it demonstrates a wide range of other biological activities, including antimicrobial, antioxidant, anti-inflammatory, antitumor, and antidepressant effects, as well as modulation of neural signaling and immune responses.^42–46^

However, natural compounds may present limitations regarding large-scale production and patentability. Thus, an alternative approach is the use of synthetic or semisynthetic compounds.^47^ In this context, carvacrol represents an attractive strategy, as it can be employed as a building block for the development of new potential drugs for various purposes.^48^ Among the compounds derived from carvacrol are the *N*-phenyl-2-phenoxyacetamides, a class of compounds that may exhibit antibacterial, antifungal, antiparasitic, anticancer, and antiviral activities.^49–52^

Thus, due to the various biological activities previously described, our group has synthesized different *N*-phenyl-2-phenoxyacetamides derived from carvacrol in a previous study.^52^ In the search for molecules with low toxicity and antiviral activity against MAYV, these compounds were evaluated for their ability to reduce viral infection *in vitro*. The compound with the highest antiviral activity was further evaluated through both *in vitro* and *in silico* assays to elucidate its mechanism of action.

## Results and Discussion

### Antiviral Screening

Since there is no specific or clinically approved antiviral agents available for alphaviruses, including MAYV,^53^ we evaluated the antiviral activity of eight *N*-phenyl-2-phenoxyacetamide derivatives of carvacrol previously synthesized by our group (identified as **1**, **3**, **4**, **5**, **6**, **7**, **9**, and **10**).^52^ Compounds were initially screened for antiviral activity against MAYV using an MTT reduction assay. For this purpose, Vero cells were infected in the presence of each compound, and cell viability was compared to that of infected but untreated cells, as well as to uninfected cells. A comparative analysis using a antiviral as a control was not performed, as our study was designed to focus on evaluating the intrinsic antiviral potential of *N*-phenyl-2-phenoxyacetamides against the Mayaro virus, without direct comparison to compounds with variable or unproven efficacy against alphaviruses. Although ribavirin, a nucleoside analog approved for use against hepatitis C virus, has demonstrated *in vitro* antiviral activity against RNA viruses and has therefore been used as a positive control in antiviral assays against MAYV,^54,55^ its activity may be inconsistent. This was shown in a study conducted by Langedries et al., in which no antiviral activity of ribavirin against MAYV was observed in experiments conducted in Vero cells.^56^

Among the compounds tested, compound **9** exhibited the highest antiviral activity against MAYV, preserving cell viability at 97.21% upon infection (Figure 1). Furthermore, in a previous study of our group, this compound showed low cytotoxicity in Vero cells, with a CC_50_ greater than 500 μM,^52^ as shown in Table 1. Although the other *N*-phenyl-2-phenoxyacetamides evaluated are structurally analogs of compound **9**, only compounds **1**, **5**, and **7** also exhibited antiviral activity against the MAYV (Figure 1). Among the compounds that demonstrated any degree of anti-MAYV activity, compound **1** exhibited the highest toxicity *in vitro*, with a CC_50_ value of 200.3 μM. In contrast, compounds **5** and **7** showed low toxicity toward mammalian cells (CC_50_ > 500 μM), but were able to preserve only 50 and 22.83% of cell viability after viral infection, respectively (Table 1). Thus, compound **9** was selected for subsequent assays.

**Figure 1:**
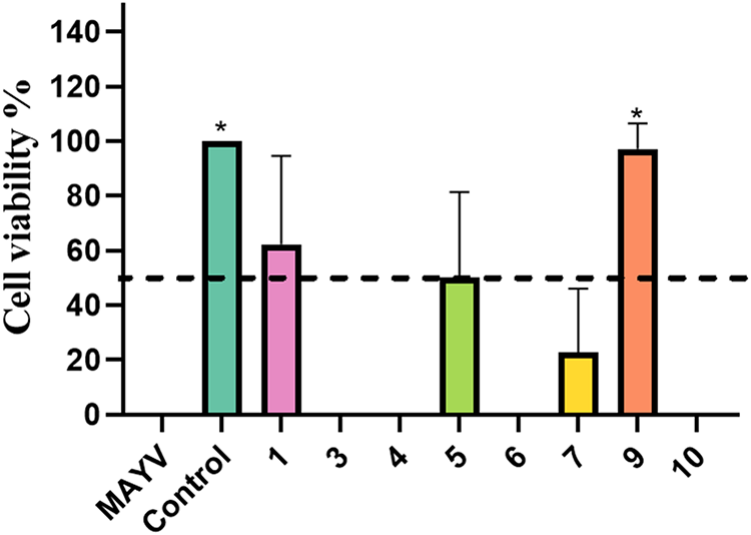
Antiviral screening of different *N*-phenyl-2-phenoxyacetamide derivatives of carvacrol against Mayaro virus. Cell viability was quantified using the MTT assay. Control: Vero cells + DMEM (100% cell viability), MAYV: Vero cells infected with MAYV (0% cell viability). Results represent the mean of three independent experiments using one-way ANOVA test. *: *p* < 0.05.

**Table 1:**
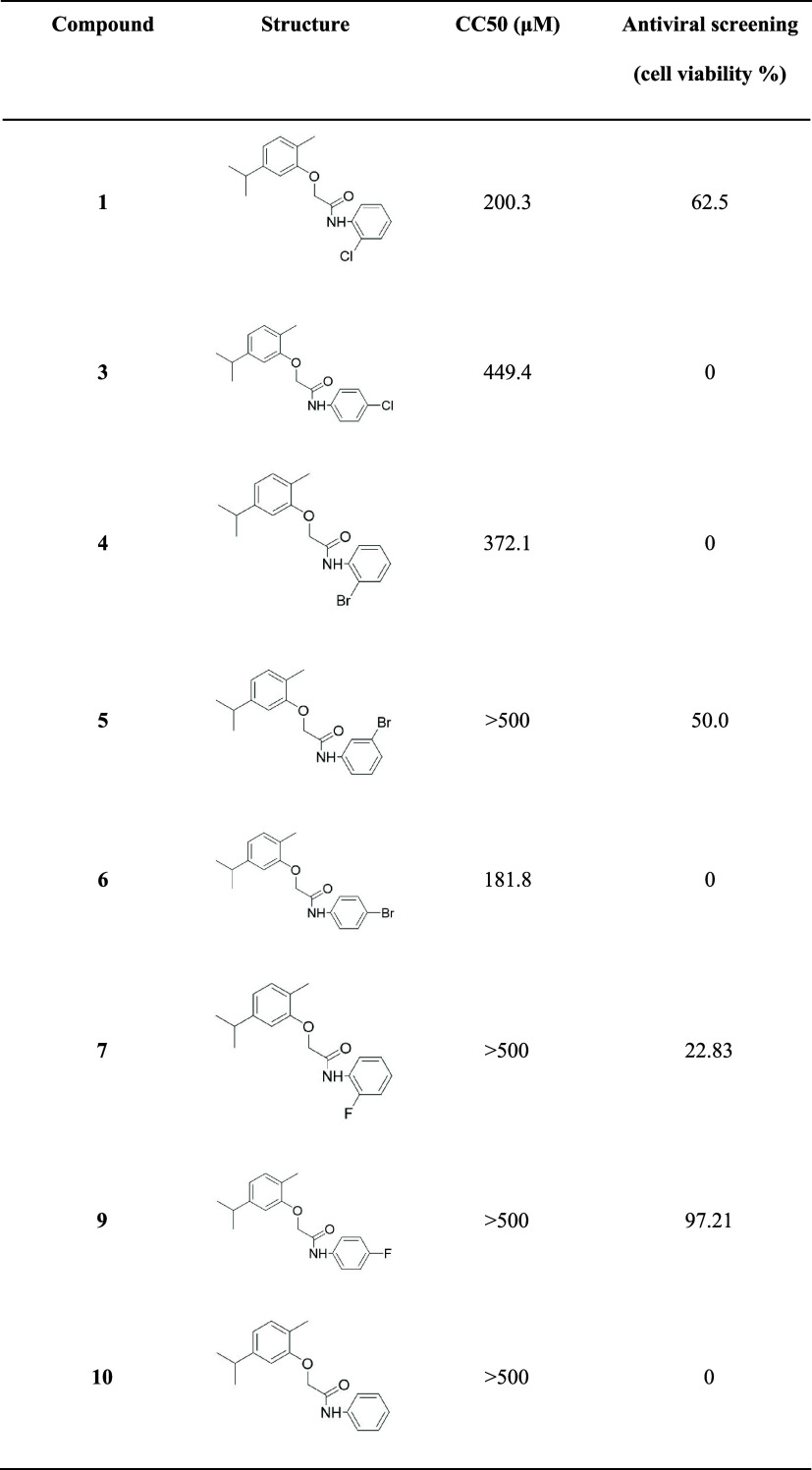
Molecular Structure of *N*-Phenyl-2-Phenoxyacetamide Derivatives of Carvacrol, Their Respective Mean Cytotoxic Concentrations (CC_50_), and Cell Survival following Treatment in the Context of MAYV Infection

### Virucidal Activity of Compound 9

Several synthetic compounds with anti-MAYV activity reported in the literature have demonstrated virucidal activity by directly interacting with the viral particle, thereby preventing or reducing the interaction of the virus with cellular receptors.^13,12,54^ Thus, we first evaluated whether compound **9** exhibits this activity against MAYV. For this purpose, Vero cells were infected with the virus previously incubated with the compound, and viral activity was assessed using a plaque reduction assay. The results obtained show that compound **9** possesses virucidal activity, causing a significant reduction in the number of lysis plaques at concentrations ranging from 500 μM to 31.25 μM, with approximately 40% reduction observed at 500 μM (Figure 2).

**Figure 2:**
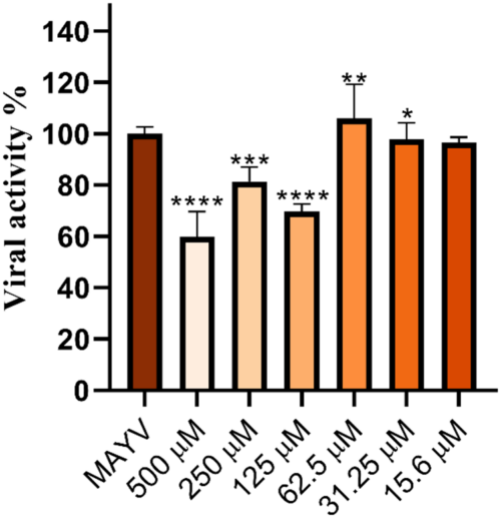
Evaluation of the virucidal activity of compound **9** against Mayaro virus. Vero cells were infected with MAYV previously incubated with different concentrations of compound **9**. MAYV: positive control consisting of Vero cells + MAYV, with the number of lysis plaques considered as 100% viral activity. Results represent the mean of three independent experiments using one-way ANOVA test. *: *p* < 0.05; **: *p* < 0.01; ***: *p* < 0.001; ****: *p* < 0.0001.

### Effect of Compound 9 on Different Stages of the MAYV Infection Cycle

To investigate the mechanism of action of compound **9**, we evaluated its effects on cellular receptors, viral adsorption, internalization, and intracellular viral activity. No reduction in the number of lysis plaques was observed when cells were pretreated with different concentrations of compound **9** (Figure 3a, pretreatment), indicating that the compound does not confer cellular protection. To assess its effect on viral adsorption, cells were maintained at 4 °C, since at this temperature the virus is capable of adsorbing but not internalizing.^57^ A reduction in viral adsorption was observed when different concentrations of the compound were tested (Figure 3b, adsorption inhibition), with an approximate 58% decrease in the number of lysis plaques at a concentration of 500 μM. Regarding internalization, a reduction of about 50% in the number of lysis plaques was also observed when concentrations between 500 μM and 62.5 μM were used (Figure 3c, internalization inhibition). When cells were treated after MAYV infection, no reduction in viral activity was observed at any of the tested concentrations (Figure 3d, post-treatment), indicating that compound **9** does not act on the stages of replication, translation and processing of viral proteins, assembly, or budding.

**Figure 3:**
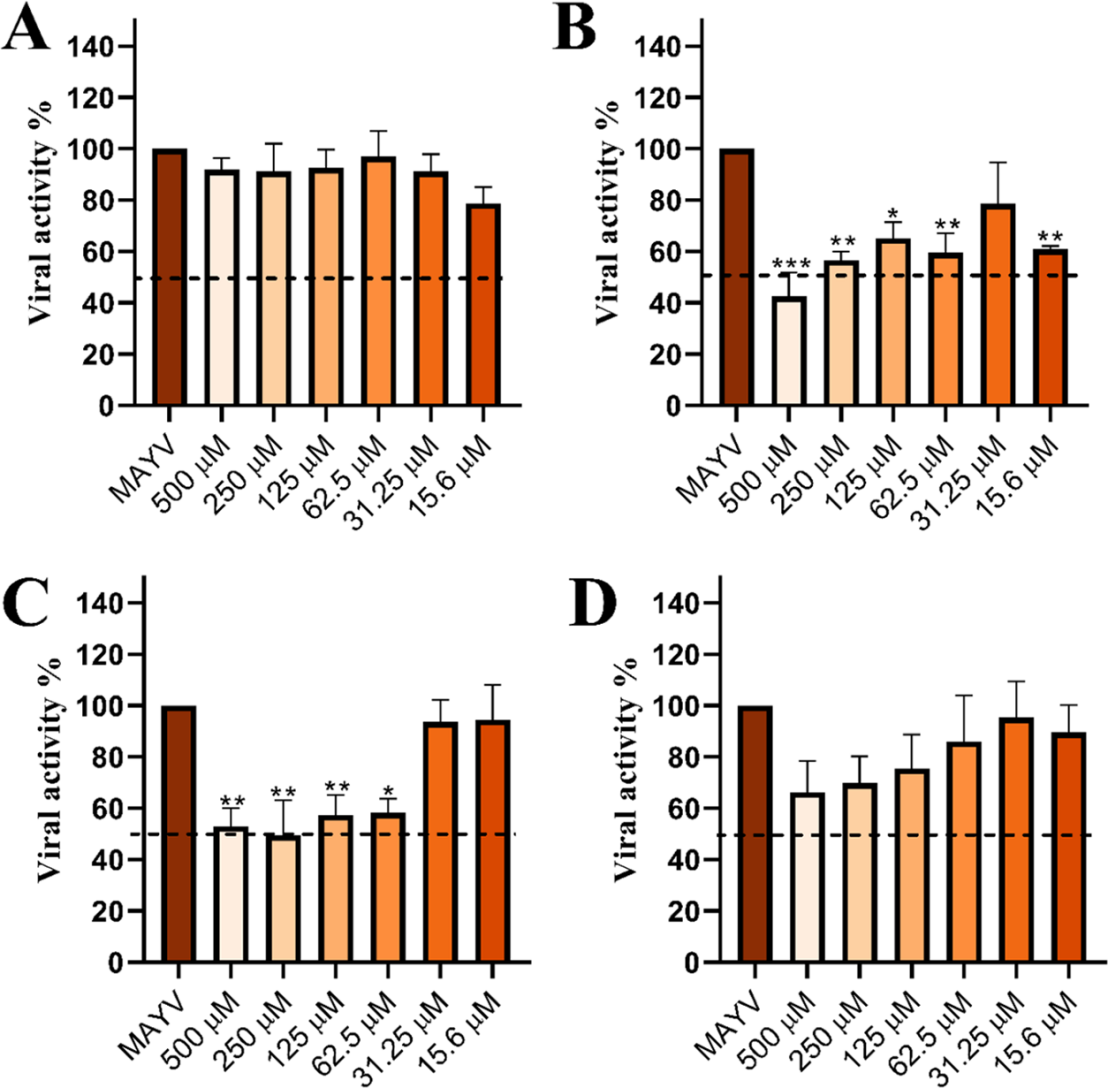
Evaluation of the mechanism of action of compound **9**. Viral activity was determined by the number of lysis plaques obtained under different conditions. (A) Pretreatment assay: Vero cells were pretreated with different concentrations of compound **9** and subsequently infected with MAYV; (B) Adsorption inhibition assay: Vero cells were infected with MAYV in the presence of different concentrations of compound **9** and maintained at 4 °C for 2 h to allow only viral adsorption; (C) Internalization inhibition assay: Vero cells were infected with MAYV, maintained at 4 °C for 2 h, then shifted to 37 °C for a brief period, and incubated with different concentrations of compound **9**; (D) Post-treatment assay: Vero cells were infected with MAYV and then treated with different concentrations of compound **9**. MAYV: positive control consisting of Vero cells + MAYV, considered as 100% viral activity. Results represent the mean of three independent experiments using one-way ANOVA test. *: *p* < 0.05; **: *p* < 0.01; ***: *p* < 0.001.

The results indicate that compound **9** directly interacts with the viral particle, which likely leads to a reduction in both viral adsorption and internalization. These actions appear to be complementary, responsible for the near 100% cell viability observed in infected cells treated with the compound during the antiviral screening assay (Figure 1). This mechanism of action differs from other compounds containing the 2-phenoxyacetamide core, which have shown antiviral activity as inhibitors of viral proteases. For example, lopinavir is a potent HIV protease inhibitor approved by the FDA for antiretroviral therapy.^58,59^ Similarly, the compound *N*-(3,4-dimethylphenyl)-2-(4-((4,6-dioxo-1,3-diphenyl-2-thioxotetrahydropyrimidin-5­(2H)-ylidene)­methyl)­phenoxyacetamide is an inhibitor of the NS2B-NS3 protease of the Zika virus.^60^ Additionally, Hariyono and colleagues, through *in silico* analyses, demonstrated the potential of different *N*-phenyl-2-phenoxyacetamides as inhibitors of the main protease M^pro^ of SARS-CoV-2.^61^ In contrast, in the present study, no reduction in viral activity was observed when the compound was added after infection (Figure 3d), suggesting that compound **9** does not interact with the MAYV protease and that the observed antiviral activity results from its action on the early stages of the viral replication cycle.

### *In Silico* Assays

The ability of compound **9** to interact with the surface glycoproteins of MAYV was evaluated through the scores generated by molecular docking using four scoring functions from the Gold software (ASP, ChemPLP, Chemscore, and Goldscore). The comparison of different scoring functions is highly recommended in various studies, as it allows for the analysis of different principles and the prediction of which interaction could be the most optimal, thereby increasing the likelihood that the interaction occurs ideally.^62–64^

The glycoproteins E1 and E2 are embedded in the viral envelope as heterodimers, which are organized into trimers to form the viral spike, where E1 and E2 traverse the lipid bilayer and interact with the C protein.^65^ Based on the data obtained from the docking of all the chains that compose the protein trimer across the four scoring functions, we can identify that, in all cases, the chains corresponding to the E2 glycoprotein presented the highest score (Table 2), indicating that the interaction of these chains with the ligand could occur with greater affinity.

**Table 2:** Molecular Docking Scores of the Six Chains Analyzed Across Each of the Four Scoring Functions, Performed Using the Gold Software

	E1 chains			E2 chains		
Scoring function	B	E	H	C	F	I
ASP	21.00	16.52	22.97	26.23	**29.09**	25.46
ChemPLP	49.65	43.63	48.73	61.13	**62.65**	51.54
ChemScore	21.27	19.11	21.21	23.08	20.40	**25.87**
GoldScore	52.23	53.38	48.53	**55.84**	55.23	49.53

Moreover, upon analyzing Figure 4, it is evident that by promoting the interaction of the ligand at the interaction center of the E2 glycoprotein chains (ASN 262), the ligand concurrently interacts with amino acid residues from both the E1 and E2 glycoproteins. This may suggest that the interaction in this region occurs with greater affinity, as indicated by the higher score, compared to the interaction of the ligand solely at the interaction center of the E1 glycoprotein (ASN 141), as shown in Table 2.

**Figure 4:**
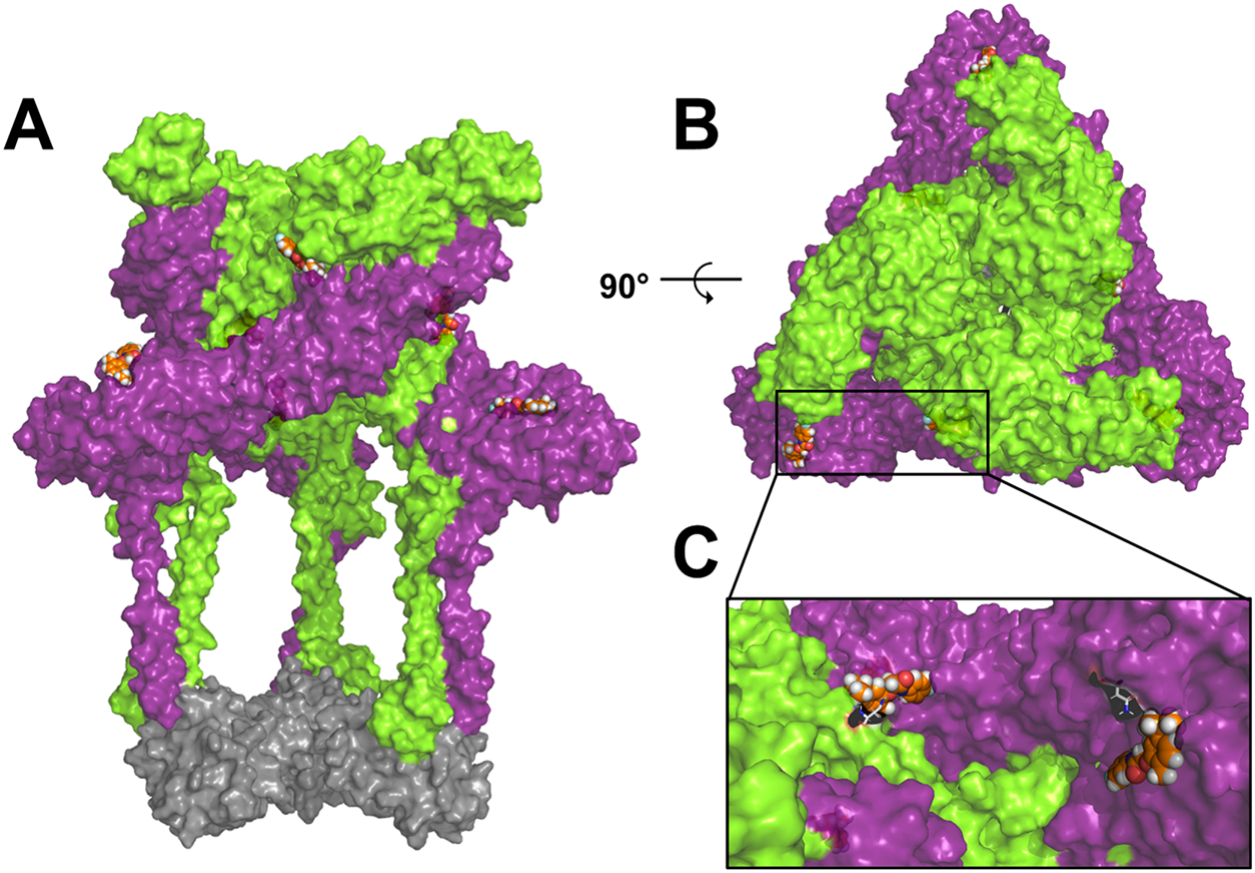
3D representation of the protein complex showing the interaction of ligand 9. Compound **9** is represented in orange spheres, with chains B, E, and H shown in purple, and chains C, F, and I shown in green, corresponding to the E1 and E2 proteins, respectively, displayed as surfaces. The chains corresponding to the capsid are represented in gray. (A) shows the frontal view of the complex with the ligands; (B) shows the top view of the complex after a 90-degree rotation; (C) represents the interaction of the ligand with the E1 and E2 chains, showing that by interacting at the active site center of E2, at residue ASN 262, the ligand also interacts concurrently with the E1 chain.

The residues ASN 141 and ASN 262 are glycosylation sites in many alphaviruses, including MAYV. These sites are important for infectivity, virulence, and viral adaptation, as they may be involved in receptor binding, particularly ASN 262.^65–67^ Thus, the interaction of compound **9** with residue ASN 262 may be related to the reduction in viral adsorption observed in the *in vitro* assays, as the interaction with the MXRA8 receptor is primarily mediated by E2, where residue ASN 262 plays a crucial role. Moreover, fusion of the viral envelope with the endosomal membrane is dependent on conformational changes in E1, which occur only after interactions between E1 and E2 are undone.^68^ By interacting with E1 and E2, compound **9** may have interfered with this process, causing a reduction in viral internalization. Therefore, the data obtained suggest that the interactions between compound **9** and the surface glycoproteins of MAYV could be responsible for the reduction in viral infectivity observed in the *in vitro* assays.

The types of bonds that occur in protein–ligand interactions are highly relevant to the affinity and stability of the interaction. In Figure 5, the 2D diagrams of the interaction between compound **9** and the chains that presented the best score in each of the analyzed scoring functions (ASP - chain F; ChemPLP - chain F; ChemScore - chain I; GoldScore - chain C) are shown. In summary, all interactions are predominantly strong bonds, with a high number of van der Waals interactions, illustrating what was reported from the analysis of the score obtained in the molecular docking. The presence of these strong bonds and stabilizing interactions would be directly related to the higher affinity and the possibility of a more stable interaction of compound **9** with the interaction site of the E2 protein. The hydrophobic interactions of the alkyl and pi-alkyl types that occur at the ends of the aromatic rings assist in the stability of the protein–ligand interaction, in addition to the noticeable and well-distributed presence of residues interacting through van der Waals forces that contribute to this stability.^67,69^

**Figure 5:**
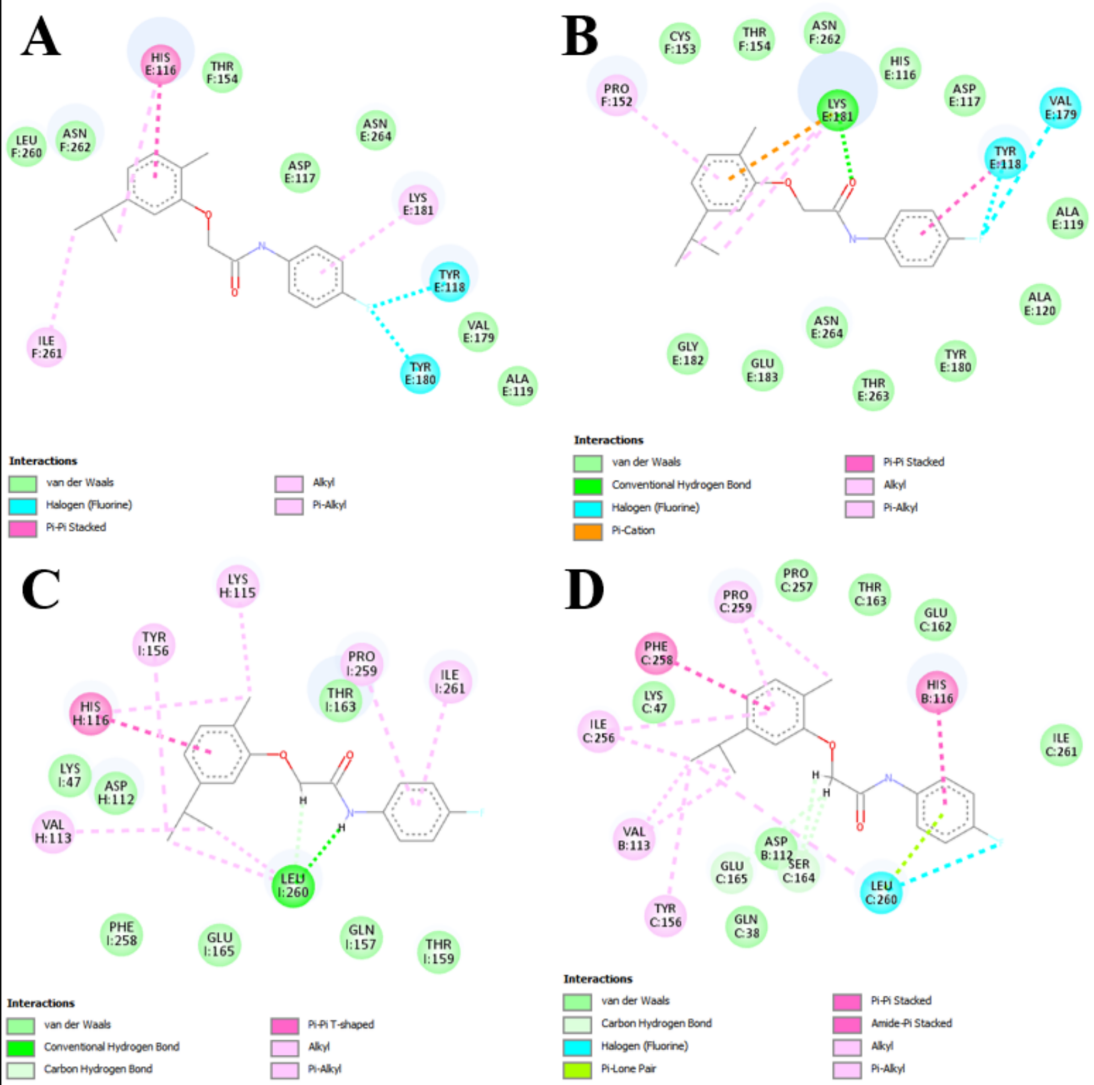
Representation of the 2D diagram of the best protein–ligand interaction in each of the four scoring functions. (A) ASP function for chain F; (B) ChemPLP function for chain F; (C) ChemScore function for chain I; and (D) GoldScore function for chain C. All possible interactions and bonds are represented, with the corresponding color legend for the bond types below each image. The predicted interactions are shown starting from the region of the compound where they occur, with the amino acid residue represented by its three-letter code and the corresponding chain.

In addition to the 2D interaction diagram presented in Figure 5, it is important to highlight that the main interactions between the protein and the ligand exhibit characteristics that render them predominantly stronger, due to the specific interaction distances between the amino acid residues and the atoms of compound **9**, as shown in Table 3.

**Table 3:** Main Bonding Interactions Observed in Chains F, F, I, and C, Which Achieved the Best Scores for Each Scoring Function (ASP, ChemPLP, ChemScore, and GoldScore, Respectively) with Ligand 9

		Binding	
Scoring function	Amino acid	Type of interaction	Distance (Å)
ASP	HIS 116	Pi-pi stacked	4.09
	TYR 118	Halogen	3.39
	TYR 180	Halogen	3.06
ChemPLP	LYS 181	H-bond	2.15
	TYR 118	Halogen	3.38
	VAL 179	Halogen	3.62
	LYS 181	Pi-cation	3.78
	TYR 118	Pi-pi stacked	4.78
ChemScore	LEU 260	H-bond	1.84
	LEU 260	Carbon H-bond	2.46
	HIS 116	Pi-pi T-shaped	4.48
GoldScore	SER 164	Carbon H-bond	2.5
	SER 164	Carbon H-bond	2.48
	SER 164	Carbon H-bond	2.23
	GLU 165	Carbon H-bond	2.68
	LEU 260	Halogen	2.57
	HIS 116	Pi-pi Stacked	3.32
	PHE 258	Amide-Pi Stacked	4.24

Pi-type interactions occurring between residues HIS 116, TYR 118, and PHE 258 and compound **9**, across the different scoring functions, reinforce the interaction strength. These interactions are within the appropriate bonding distances and contribute significantly to bond strength, complex stabilization, binding affinity, and overall architecture.^67,69^

Nonconventional hydrogen bonds (carbon H-bonds), observed with residues LEU 260 and SER 164, due to their longer bond distances, are classified as weaker interactions. Nevertheless, they assist in stabilizing the complex and contribute to the overall protein–ligand binding affinity.^70^

The structural variation among the analyzed compounds is restricted to the presence of halogen substituents (Br, Cl, or F) and their positions at ortho, meta, or para (compounds **1**, **3**, **4**, **5**, **6**, and **7**), or to the absence of a substituent (compound **10**). In the *in vitro* assays, a significant difference in antiviral activity was observed between the evaluated compounds and compound **9**. Therefore, an *in silico* comparison was also performed to evaluate how halogen substitution, or its absence, could be relevant through molecular docking prediction. Among the scoring functions available in the GOLD software, the most appropriate for assessing the importance of the halogen in the ligand–protein interaction was the ChemPLP function, which is currently the default scoring function in GOLD due to its higher average performance compared to other functions and its more robust characteristics for predicting protein–ligand interactions.^64^

In the molecular docking analysis using GOLD with the ChemPLP scoring function, chain F of the E2 protein was considered, since it had shown the highest score in the preliminary analysis performed only with compound **9** (Table 2). All analyzed compounds presented lower scores than the interaction of the same chain F with compound **9**, as shown in Table 4. These *in silico* results corroborate the *in vitro* findings, as the other compounds did not show significant antiviral activity against MAYV. Moreover, the docking scores indicate that, despite the high structural similarity among the compounds, differing only in the type and position of the substituent, the presence of fluorine at the para position in compound **9** was decisive for the observed antiviral activity and likely for the interaction with the viral protein.

**Table 4:** Molecular Docking Scores for Chain F Analyzed Across Eight Compounds, Calculated Using GOLD Software with the ChemPLP Scoring Function

Compounds	1	3	4	5	6	7	9	10
Score	55.58	62.17	54.84	58.52	62.24	56.74	62.65	56.65

As illustrated in the 2D diagram (Figure 6), the region where fluorine is present in compound **9** exhibits stronger interactions, such as halogen bonding, which do not occur significantly in compounds with chlorine or bromine substituents. Even compound **7**, which also contains fluorine but at the ortho position, did not show a favorable interaction with the E2 protein. Although compounds **3** and **6** bear substituents at the para position and exhibited higher scores than the other compounds, their values were still lower than those of compound **9**, reinforcing that chlorine and bromine substituents do not promote strong interactions with the protein.

**Figure 6:**
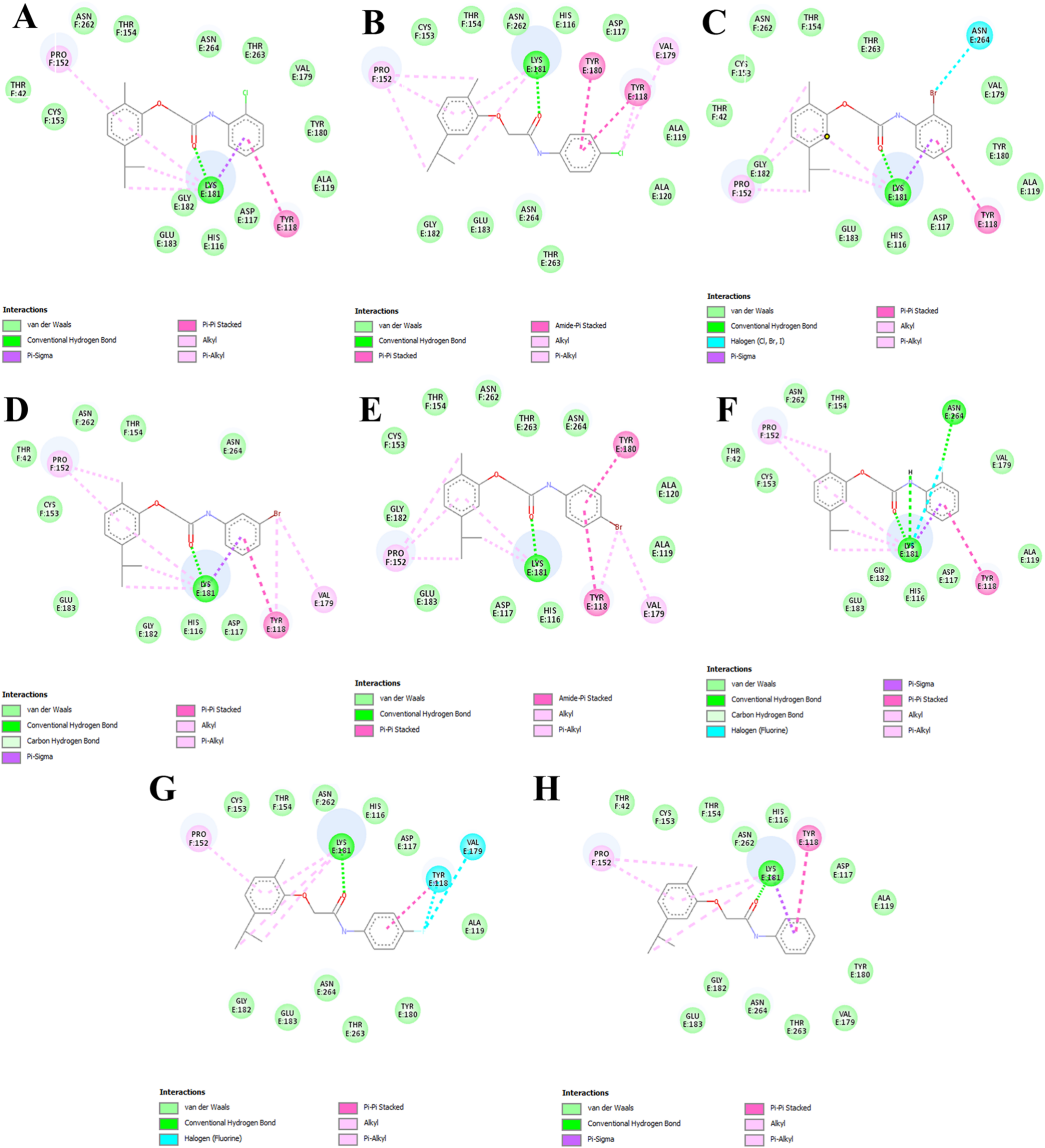
Representation of the 2D diagram of protein–ligand interactions for the eight compounds with chain F of the E2 protein, based on the ChemPLP scoring function from the GOLD software. Each diagram shows the interaction of one compound: (A) **1**, (B) **3**, (C) **4**, (D) **5**, (E) **6**, (F) **7**, (G) **9**, and (H) **10**. All possible interactions and bonds are represented, with the corresponding color legend for the bond types below each image. The predicted interactions are shown starting from the region of the compound where they occur, with the amino acid residue represented by its three-letter code and the corresponding chain.

It is important to highlight that the interaction regions with the E1 (ASN 141) and E2 (ASN 262) chains identified *in* *silico* are conserved between the Mayaro virus and the Chikungunya virus,^65^ ,^71^ suggesting that compound **9** may also exhibit antiviral activity against CHIKV. Given the similarity of symptoms in patients infected by these viruses and their cocirculation in various regions,^72^ an antiviral compound that could be used in the treatment of both diseases would be highly valuable. Therefore, further studies are necessary to evaluate the activity of compound **9** as an antiviral agent against CHIKV.

Thus, the results obtained demonstrate the importance of *N*-phenyl-2-phenoxyacetamides as a new class of compounds with antiviral activity against MAYV, paving the way for potential therapeutic applications. In this context, compound **9** proved to be promising due to its pronounced antiviral activity and low toxicity in mammalian cells. Therefore, the efficacy and safety of this compound should be evaluated through *in vivo* assays. Given its potential as a broad-spectrum antiviral, this *N*-phenyl-2-phenoxyacetamide emerges as a promising candidate for the development of innovative strategies to combat arthritogenic alphaviruses, such as MAYV and CHIKV.

## Conclusion

This study is the first to demonstrate the antiviral activity of *N*-phenyl-2-phenoxyacetamides against Mayaro virus (MAYV). Among the eight molecules evaluated, compound **9** exhibited the strongest antiviral activity by directly biding to viral particles, thereby reducing adsorption and internalization possibly through interactions with glycoproteins E1 and E2. This observation is supported by *in silico* analyses, which show the compound’s ability to interact with the regions of the viral particle involved in binding to the MXRA8 cellular receptor and the viral fusion process.

## Methods

### Cell Lines and Viral Stock

Vero cells (ATCC CCL-81) were used in all *in vitro* assays. Cells were cultured in DMEM medium supplemented with 2% fetal bovine serum (FBS), penicillin, and streptomycin, and incubated at a constant temperature of 37 °C under a controlled atmosphere of 5% CO_2_. Mayaro virus (ATCC VR 66, strain TR 4675) was initially propagated in C6/36 cells cultured in L-15 medium supplemented with 2% FBS, incubated at 28 °C. For viral titer determination, Vero cells (10^5^ cells/well) were seeded in 24-well plates and cultured for approximately 24 h to achieve 80% confluence. Subsequently, the culture medium was replaced with 10-fold serial dilutions of the viral aliquot. The plate was maintained under constant agitation for 1 h to facilitate viral adsorption. The medium was then replaced with a semisolid medium (incomplete DMEM and 1% carboxymethylcellulose at a 2:1 ratio). Plates were incubated for 48 h under the aforementioned conditions, and cytopathic effects were monitored. After this period, cells were fixed with 10% formaldehyde and stained with 5% crystal violet solution for plaque counting. The mean number of plaques was used to determine the number of plaque-forming units (PFU), and the viral titer was expressed as PFU/mL.

### *N*-Phenyl-2-Phenoxiacetamides

Eight *N*-phenyl-2-phenoxyacetamides derived from carvacrol, identified as compounds **1**, **3**, **4**, **5**, **6**, **7**, **9**, and **10**, were previously synthesized and characterized by our group. Briefly, carvacroxyacetic acid was synthesized from carvacrol and used as the starting material for the preparation of the various *N*-phenyl-2-carvacroxyacetamides via Steglich amidation (DCC/DMAP) with anilines substituted at the ortho, meta, or para positions by Cl, Br, or F.^52^ The structures and respective CC_50_ values in Vero cells are shown in Table 1.

### Antiviral Screening Assay

To evaluate the antiviral potential of the molecules under study, an MTT (3-(4,5-dimethylthiazol-2-yl)-2,5-diphenyltetrazolium bromide) reduction assay was employed. Vero cells were seeded in 96-well plates (10^4^ cells/well) and cultured until reaching approximately 80% confluence. Cells were infected with MAYV (MOI 1) previously treated separately with the different compounds for 1 h at 37 °C, at concentrations determined based on each compound’s CC_50_. For compounds with undetermined CC_50_ values, a concentration of 500 μM (the highest concentration tested in the previous study^52^) was used. After incubation, the viral suspension was removed, DMEM medium was added, and cells were incubated for 48 h at 37 °C under a 5% CO_2_ atmosphere. Cell viability was determined by absorbance reading at 540 nm after solubilization of formazan crystals with DMSO, with the positive control (Vero cells infected with MAYV) defined as 0% cell viability and the negative control (uninfected Vero cells) defined as 100% cell viability. The compound providing the greatest cellular protection was selected for subsequent assays.

### Virucidal Assay

To determine the action of the molecule on viral particles, Vero cells cultured in 24-well plates (10^5^ cells/well) at 80% confluence were infected with MAYV (100 PFU/well) previously incubated for 1 h with different concentrations of the compound at 37 °C (100 μL/well). Plates were maintained under agitation for 1 h, and afterward, the viral suspension was replaced with 1 mL of semisolid medium (DMEM + 1% carboxymethylcellulose). Plates were incubated for 48 h at 37 °C and 5% CO_2_ and subsequently fixed with 10% formaldehyde followed by staining with 5% crystal violet solution for plaque counting. DMEM was used as the negative control, and 100 PFU of untreated MAYV was used as the positive control.

### Mechanism of Action Assays

Plaque reduction assays were performed under different conditions to evaluate whether the antiviral activity exhibited by compound 9 results from interactions with the viral particle or the host cell, according to previously described methods^12,57^ with minor modifications.

### Pretreatment Assay

To determine whether the compound exerts a protective effect on cells prior to viral infection, Vero cells cultured in 24-well plates (10^5^ cells/well) were treated for 3 h at 37 °C with 100 μL of the compound at different concentrations, with periodic agitation. Subsequently, the medium was replaced with 100 μL of a solution containing MAYV (100 PFU), and the plates were incubated under constant agitation for 1 h. The viral suspension was then replaced with a semisolid medium (DMEM + 1% carboxymethylcellulose), and the plates were incubated for 48 h at 37 °C under a 5% CO_2_ atmosphere. Cells were fixed with 10% formaldehyde and stained with 5% crystal violet solution for plaque counting. DMEM was used as the negative control, and 100 PFU of MAYV was used as the positive control (untreated).

### Post-Treatment Assay

To determine whether the compound acts after viral infection, Vero cells cultured in 24-well plates (10^5^ cells/well) were initially infected with 100 μL of MAYV (100 PFU) and incubated under constant agitation for 1 h. The viral suspension was removed and replaced with 100 μL of the compound at different concentrations, followed by incubation for 3 h at 37 °C with periodic agitation. Subsequently, the medium was replaced with semisolid medium (DMEM + 1% carboxymethylcellulose). After 48 h of incubation at 37 °C and 5% CO_2_, the cells were fixed with 10% formaldehyde and stained with 5% crystal violet for plaque counting. For the negative control, DMEM was used, and for the positive control, 100 PFU of MAYV (untreated).

### Adsorption Inhibition Assay

To assess whether the compound interferes with the viral adsorption step, 24-well plates (10^5^ cells/well) were cooled at 4 °C for 30 min. The culture medium was removed, and cells were washed with cold PBS, followed by the addition of 100 μL of a solution containing MAYV (100 PFU) and different concentrations of the compound. Cells were incubated at 4 °C for 2 h and subsequently washed with cold citrate buffer (0.1 M citric acid and 0.1 M sodium citrate) and PBS. Semisolid medium (DMEM + 1% carboxymethylcellulose) was then added, and plates were incubated at 37 °C for 48 h. Cells were fixed and stained for plaque counting as previously described. DMEM was used as the negative control, and 100 PFU of MAYV served as the positive control (untreated).

### Internalization Inhibition Assay

To evaluate whether the compound affects the internalization step, 24-well plates (10^5^ cells/well) were cooled at 4 °C for 30 min. The culture medium was removed, and cells were incubated with MAYV (100 PFU/well) at 4 °C for 2 h. Cells were washed with cold PBS, and 1 mL of DMEM was added followed by incubation for 5 min at 37 °C. Subsequently, different concentrations of the compound were added and incubated for 1 h at 37 °C. Cells were then washed with cold PBS and citrate buffer. Finally, semisolid medium (DMEM + 1% carboxymethylcellulose) was added, and plates were incubated at 37 °C for 48 h. Cells were fixed and stained for plaque counting. DMEM was used as the negative control, and 100 PFU of MAYV was used as the positive control (untreated).

### *In Silico* Assays

The protein complex analyzed was selected from the RCSB Protein Data Bank (PDB accession number: 7KO8). The selected complex exhibits a trimeric structure, composed of the capsid protein (C), glycoprotein E1 (E1), and glycoprotein E2 (E2). Only chains B, E, and H (from E1) and chains C, F, and I (from E2) were used as docking targets. The interaction site was defined with a radius of 10 Å for protein–ligand interactions using the GOLD software. For docking, all cocrystallized ligands were removed, and all necessary hydrogens were added to the proteins to enable proper protein–ligand interaction. The central residues selected for interaction were asparagine 141 (ASN 141) in E1 and asparagine 262 (ASN 262) in E2, both identified as key residues involved in the interaction with the host receptor MXRA8.^65^

A standardization was performed for each of the E1 and E2 proteins, using the first chain of each (chains B and C, respectively), to determine the number of conformations and the ligand orientations that would yield the highest scores across the different scoring functions. The corresponding additional chains for each protein followed the established standardization protocol. Considering the distinct scoring principles employed by each scoring function in the GOLD software, all four scoring functions were used to compare the best protein–ligand interactions based on the final docking scores.

Two-dimensional diagrams of protein–ligand interactions were generated using Discovery Studio, and three-dimensional interaction images were created using PyMOL.

### Statistical Analysis

Statistical analyses were performed using GraphPad Prism 8 software. Comparisons between treatments and controls were conducted using one-way ANOVA with multiple comparisons. All data represent the mean of three independent experiments, each performed in triplicate.
